# Explicit attention interferes with selective emotion processing in human extrastriate cortex

**DOI:** 10.1186/1471-2202-8-16

**Published:** 2007-02-22

**Authors:** Harald T Schupp, Jessica Stockburger, Florian Bublatzky, Markus Junghöfer, Almut I Weike, Alfons O Hamm

**Affiliations:** 1 Department of Psychology, University of Konstanz, Germany; 2 Institute for Biomagnetism and Biosignalanalysis, Münster University Hospital, Germany; 3 Department of Psychology, University of Greifswald, Germany

## Abstract

**Background:**

Brain imaging and event-related potential studies provide strong evidence that emotional stimuli guide selective attention in visual processing. A reflection of the emotional attention capture is the increased Early Posterior Negativity (EPN) for pleasant and unpleasant compared to neutral images (~150–300 ms poststimulus). The present study explored whether this early emotion discrimination reflects an automatic phenomenon or is subject to interference by competing processing demands. Thus, emotional processing was assessed while participants performed a concurrent feature-based attention task varying in processing demands.

**Results:**

Participants successfully performed the primary visual attention task as revealed by behavioral performance and selected event-related potential components (Selection Negativity and P3b). Replicating previous results, emotional modulation of the EPN was observed in a task condition with low processing demands. In contrast, pleasant and unpleasant pictures failed to elicit increased EPN amplitudes compared to neutral images in more difficult explicit attention task conditions. Further analyses determined that even the processing of pleasant and unpleasant pictures high in emotional arousal is subject to interference in experimental conditions with high task demand. Taken together, performing demanding feature-based counting tasks interfered with differential emotion processing indexed by the EPN.

**Conclusion:**

The present findings demonstrate that taxing processing resources by a competing primary visual attention task markedly attenuated the early discrimination of emotional from neutral picture contents. Thus, these results provide further empirical support for an interference account of the emotion-attention interaction under conditions of competition. Previous studies revealed the interference of selective emotion processing when attentional resources were directed to locations of explicitly task-relevant stimuli. The present data suggest that interference of emotion processing by competing task demands is a more general phenomenon extending to the domain of feature-based attention. Furthermore, the results are inconsistent with the notion of effortlessness, i.e., early emotion discrimination despite concurrent task demands. These findings implicate to assess the presumed automatic nature of emotion processing at the level of specific aspects rather than considering automaticity as an all-or-none phenomenon.

## Background

Emotional cues guide selective visual attention and receive enhanced processing [1-4]. Functional Magnetic Resonance Imaging (fMRI) revealed increased BOLD (Blood Oxygen Level Dependent) signals in associative visual regions (extrastriate, occipito-parietal, and inferior temporal cortex) and subcortical limbic structures when viewing emotionally arousing compared to neutral pictures [e.g., [5-7]]. A recent fMRI study determined that increased activity to emotional stimuli in the lateral inferior occipital and inferior temporal visual cortex is retained when the stimulus materials were shown at rapid presentation rates (3 and 6 Hz). Thus, even when neuronal processing is limited by short exposure times and conceptual masking, as each picture is replaced by a new semantically unrelated image, associative visual areas are selectively activated by emotional cues [8]. Event-related brain potential (ERP) studies detailed the temporal dynamics of emotion processing in the visual cortex. Using rapid presentation rates, a difference in processing emotional (pleasant and unpleasant) compared to neutral pictures is reflected by an enhanced negativity over temporo-occipital sites. This Early Posterior Negativity (EPN) develops around 150 ms after stimulus onset and lasts until about 300 ms. Estimates of the generator sources of the differential ERP activity suggested that emotional stimuli receive enhanced processing in occipito-temporo-parietal regions, particularly pronounced for right hemispheric regions [9-12]. Recent studies extended these findings to other emotional stimulus materials such as faces, words, or even affective hand gestures [13,14]. Accordingly, the EPN was suggested to reflect the facilitated encoding of visual scenes depicting information of emotional significance.

Functional and evolutionary considerations suggest the benefit of paying attention to the good and bad things in the environment. In a world where various stimuli compete for attentional resources, the fast and reliable detection of positive and negative reinforcers facilitates adaptive behavior, finally promoting survival and reproductive success [1,2]. From this point-of-view, it is of considerable interest to determine to what extent emotional stimuli are processed automatically [cf. [15,16]]. Focusing specifically on the early selective emotion processing indexed by the EPN, some aspects of automaticity have been recently explored. For instance, the EPN effect occurs spontaneously in passive viewing conditions, while participants hold unrelated task goals in mind, and does not depend on the novelty of the stimulus materials [17,18]. These results suggest that the emotional EPN modulation reflects an automatic process in the sense of being unintentional, autonomous, and involuntary. However, because automaticity cannot be captured as an all-or-none phenomenon, the individual aspects associated with automaticity need to be explored separately [19]. One further aspect of automaticity is the notion of effortlessness suggesting that the preferential processing of emotional cues is not consumptive of limited processing resources and will operate even when attentional resources are scarce. Challenging the notion of effortlessness, recent fMRI- and ERP studies demonstrated the interference of selective emotion processing when attention was manipulated in the spatial domain [20]. For instance, participants had to discriminate the orientation of eccentrically presented bars while maintaining fixation on centrally presented emotional or neutral faces. Under task load, as compared with control conditions, processing of emotional compared to neutral faces was not associated with increased activation in associative visual processing areas or amygdala [[21], see also [22]]. Complementary evidence was provided by recent ERP research. For instance, it was observed that task-relevant bar stimuli cued by fearful rather than neutral faces were associated with increased P1 components over lateral occipital leads [23]. In addition, a reduced N1 peak over frontal sites to fearful as compared to neutral faces was observed when the faces were presented at attended locations while being absent when presented at non-attended locations [24]. These findings suggest an interference of emotion processing with primary attention tasks. However, since different mechanisms of attentional selection exist [3], it appears interesting to determine whether findings observed in the domain of spatial attention extend to feature-based attention.

**Figure 1:**
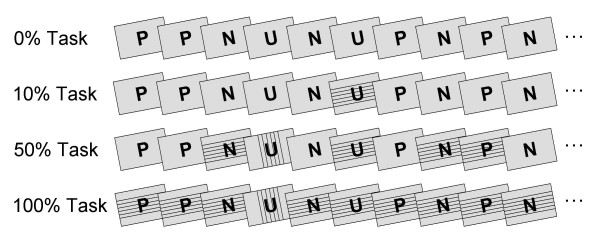
**Schematic illustration of the four experimental conditions**. In one condition, participants passively viewed the pictures (0 % task). Three further conditions included a feature-based counting task. Task-related stimuli were created by overlaying 6 thin horizontal or vertical lines. The lines superimposed on the IAPS pictures covered approximately 6 % of the picture and did not comprise the perceptibility of the IAPS pictures. The probability of task-related stimuli was varied across the three task conditions including 10 %, 50 %, or 100 % of trials (for the last two rows the fourth trial in the shown example is one of the rare target trials). In all conditions, pictures were presented as rapid and continuous stream. Abbreviations P, N, U refer to Pleasant, Neutral, and Unpleasant picture contents.

The present study assessed the modulation of the rapid emotion discrimination indexed by the EPN in the context of a primary feature-based counting task. As in previous studies, emotional and neutral stimuli were drawn from the International Affective Picture Series (IAPS) [25] presented as rapid (3 Hz) and continuous stream of pictures. To implement a concurrent feature-based counting task, task-related stimuli were created by overlaying thin lines on the IAPS images (see Figure 1). In each of the task conditions, participants' task was to silently count stimuli containing the rare vertical or horizontal target orientation (balanced across participants). The demand of the primary attention task was systematically increased by varying the proportion of task-relevant stimuli in the stream of IAPS pictures across three experimental conditions (10 %, 50 % or 100 % probability, respectively). In an additional condition (0 % task), IAPS pictures were presented without any task-relevant stimuli to replicate previous studies in which the pictures were passively viewed. The order of the four experimental conditions was balanced across participants. A first set of analyses focused on the explicit selective attention task using behavioral performance and selected ERP components as dependent measures to assure that participants conducted the task appropriately. A second set of analyses determined whether selective emotion processing as indexed by the EPN is subject to interference by a competing attention task or reflects an automatic phenomenon. The interference account predicts that the emotional EPN modulation is attenuated or abolished with increasing task demands. The notion of automaticity implies that the emotional modulation is independent from processing resources devoted to the primary attention task and therefore predicts augmented EPN amplitudes to emotional cues across all task conditions.

## Results and discussion

### Explicit attention task: behavioral and ERP results

Performance in the primary feature-based counting task was captured by an overall error score calculated separately for each of the three task conditions. Overall, behavioral performance was good with an average of 7.4 % errors across the three task conditions. Furthermore, repeated measure ANOVA (analysis of variance) indicated that performance was comparable across task conditions (M_10% _= 7.1, SD = 11.7; M_50% _= 5.8, SD = 10.6; M_100% _= 8.7, SD = 7.9), Task F(2,30) < 1, ns.

Participants' ratings of the difficulty to perform the experimental task indicated that task demand varied across experimental conditions (M_0% _= 26.9, SD = 28.9; M_10% _= 43.1, SD = 21.5; M_50% _= 61.3, SD = 18.9; M_100% _= 79.4, SD = 12.4), Task F(3,45) = 16.0, p < .0001, Greenhouse-Geisser epsilon = 0.75. While 0 % and 10 % task conditions did not significantly differ, both conditions were perceived as less difficult compared to the 50 % and 100 % task conditions, ts(15) > 2.7, p < .05. In addition, the 100 % condition was perceived as more demanding compared to the 50 % task condition t(15) = 3.1, p < .05.

Previous ERP studies established reliable ERP indices of feature-based selective attention. Occurring around 150–350 ms poststimulus, selective attention to specific stimulus features (i.e., shape, form, color) and higher order semantic categories is revealed by a more pronounced negativity over temporo-occipital sensor regions (labeled as Selection Negativity (SN), N2, or Posterior Negativity) [26-29]. The 50 % and 100 % task conditions provided a sufficient number of target trials for the reliable assessment of the SN component, which was submitted in separate analyses to repeated measures ANOVA including factors of Target (target vs. non-target) and Laterality (left and right). Analyses revealed significantly increased SN amplitudes for target compared to non-target stimuli in both task conditions (see Table 1), Target F_50%_(1,15) = 5.2, p < .05, F_100%_(1,15) = 22.9, p < .001. No significant effects involving laterality were observed in these analyses.

**Table 1:** ERP components of selective attention

	Non-Target		Target	
Measure	Left	Right	Left	Right

SN				
50 % task	0.4	0.4	-0.3	-0.2
	(0.9)	(1.0)	(1.4)	(1.5)
100 % task	1.2	1.4	0.0	0.0
	(0.9)	(1.1)	(1.6)	(1.6)
P3b				
50 % task	0.1	0.1	2.9	2.5
	(0.7)	(0.7)	(2.7)	(1.9)
100 % task	0.0	0.0	2.1	2.0
	(1.0)	(0.7)	(1.4)	(1.5)

Mean (SD) amplitude in microvolts for the Selection Negativity and P3b components during processing of target and non-target pictures separately for 50 % and 100 % task conditions and left and right hemispheric regions.

Another hallmark finding in ERP studies of selective attention is that target compared to non-target processing is reflected by increased P3b amplitudes, i.e., a positive potential shift over centro-parietal sensor regions around 300–700 ms poststimulus [30]. Separate repeated measures ANOVA including factors of Target and Laterality revealed enlarged P3b potentials for target compared to non-target stimuli, Target F_50%_(1,15) = 31.3, p < .001, F_100%_(1,15) = 46.9, p < .001. Again, no significant effects of hemispheric differences were observed.

Taken together, behavioral and ERP measurements provide evidence that the participants complied with the instruction to perform the feature-based counting task. Replicating previous findings of feature-based selective attention, increased SN and P3b amplitudes were observed for target compared to non-target stimuli in the 50 % and 100 % task conditions. In addition, behavioral performance appeared good across all task conditions. Behavioral task demand has been highlighted as an important factor to account for differences in findings across studies exploring selective emotion processing while participants performed primary spatial attention tasks [20]. It was suggested that interference with selective emotion processing varies with the extent to which the explicit attention task demands resources and is most clearly obtained with highly demanding tasks. In regard of previous studies, the comparably low rate of errors in the present study suggests that the counting task was moderately taxing resources. However, while presumably not exhausting processing resources, ratings of difficulty demonstrate that both, the 50 % and 100 % task conditions were more demanding compared to passive viewing and 10 % task condition. Furthermore, cognitive and neuroimaging studies revealed the interference of distractor processing by increasing task demands in the perceptual domain [31,32]. Considered from the perspective of the load theory of attention [32], the present attention task taxed resources in the perceptual and cognitive domain by requiring processes of stimulus categorization and working memory. The higher probability of task-related stimuli in the 50 % and 100 % task condition presumably increased both perceptual and working memory load. Thus, while the concurrent attention task enabled to examine the hypothesis whether selective emotion processing operates automatically or is dependent on limited processing resources, the study is limited with regard to isolating task load in distinct sub-processes.

### The interaction of emotion and attention

The interaction of emotion and explicit attention was assessed by submitting the EPN amplitude to a repeated measure ANOVA including Valence (pleasant, neutral, unpleasant pictures), Task (0 %, 10 %, 50 %, 100 % probability of task-relevant stimuli), and Laterality (left, right hemisphere). The present data replicated previous findings regarding the modulation of EPN as a function of emotional content, Valence F(2,30) = 24.6, corrected p < .0001, Greenhouse-Geisser epsilon = 0.77. Pleasant and unpleasant pictures were associated with increased EPN amplitudes compared to neutral contents, ts(15) = -6.1 and -3.9, p < .01, respectively. In addition, as in previous studies [17,18], pleasant pictures elicited greater EPN amplitudes than unpleasant contents, t(15) = -3.9, p < .01.

**Figure 2:**
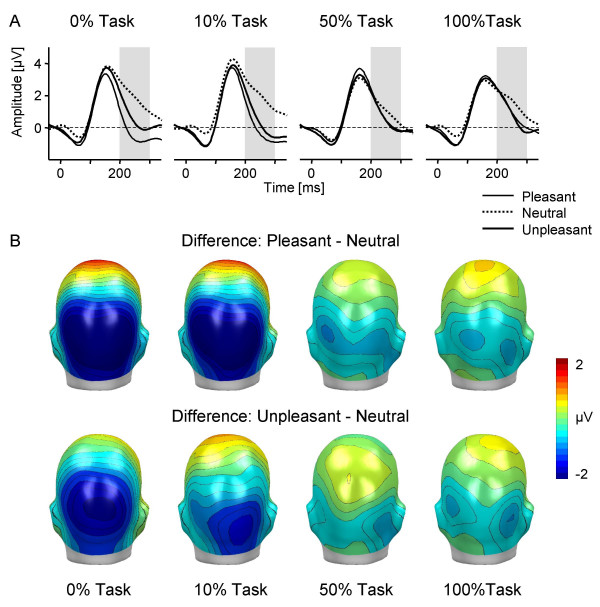
**Interference of selective emotion processing by task demand**. (A) Grand-averaged ERP waveforms elicited by pleasant, neutral, and unpleasant pictures in the four experimental conditions for a right occipital sensor. The grey-shaded area refers to the analyzed EPN time interval from 200–300 ms. (B) Topographical difference maps for pleasant-neutral and unpleasant-neutral in the four conditions projected on the back view of a model head (mean from 200–300 ms).

Of main interest, interference of selective emotion processing with the explicit attention task was indicated by the significant interaction of Valence and Task, F(6,90) = 10.4, corrected p < .0001, Greenhouse-Geisser epsilon = 0.73. The interaction is illustrated in Figure 2 by displaying ERP waveforms of a right hemispheric occipital sensor and back views of the mean topographical ERP difference maps (emotional – neutral picture contents). Obviously, emotional modulation appeared pronounced for the 0 % and 10 % task condition, while being greatly attenuated for the 50 % and 100 % tasks. Accordingly, to follow up the interaction of Valence by Task, emotional modulation was determined for each task condition separately. Both, the 0 % and 10 % task conditions revealed highly significant main effects of Valence, Fs(2,30) = 28.1 and 35.1, corrected ps < .0001, Greenhouse-Geisser epsilon = 0.87 and 0.91; indicating enlarged EPN amplitudes for pleasant and unpleasant compared to neutral images, ts(15) < -5.6, p < .0001. In contrast, separate analyses of the higher demanding 50 % and 100 % conditions revealed no significant effect of Valence, Fs(2,30) = 2.6 and 1.5, ns., Greenhouse-Geisser epsilon = 0.89 and 0.92.

A caveat with regard to the interference account is that the superposition of lines masked the IAPS stimulus materials, which might have prevented the observation of the emotional modulation indicated by the EPN. One possibility to explore this issue is to limit the analyses of the 50 % task condition to trials in which the IAPS pictures were presented without superimposed lines. Providing additional support for the interference account, ANOVA analysis of the 50 % task confined to IAPS pictures without lines revealed no EPN differentiation of emotional compared to neutral picture contents, Valence, F(2,30) = 1.1, ns., Greenhouse-Geisser epsilon = 0.94. Furthermore, 3 (Valence) × 2 (Task) ANOVAs were calculated comparing IAPS pictures of the 50% condition without superimposed lines with either passive viewing or the 10 % task condition. Both analyses confirmed the interference of selective emotion processing with the explicit attention task reported above, Valence × Task, Fs(2,30) = 14.0 and 12.2, corrected p < .0001, Greenhouse-Geisser epsilon = 0.94 and 0.99, respectively. Difference scores of the EPN between emotional (pleasant and unpleasant) and neutral pictures were calculated to further demonstrate the interference of emotion processing in the 50 % task condition. Repeated measures ANOVA revealed that the emotional differentiation of the EPN was significantly attenuated in the 50 % task condition compared to the passive viewing and 10 % task conditions, Task Fs(1,15) = 18.1 and 14.7, p < .0001. In contrast, within the demanding task conditions (50 % task without lines, 50 % task with lines, 100 % task), the difference score was comparable, Task F(2,30) = 0.1, ns. Taken together, the notion that the pattern of results observed in the present study is attributable to impoverished stimulus perceptibility in the 50 % and 100 % task conditions received no empirical support.

Overall, the novel finding of the present study is that the selective processing of emotional cues as indexed by the EPN was pronouncedly attenuated while participants performed demanding attention tasks. In contrast, passive picture viewing without counting task replicated previous findings in that emotional images were associated with enlarged EPN amplitudes compared to neutral cues [9-11]. Furthermore, results of the 10 % condition replicated previous findings in that holding a task set in mind did not attenuate emotional modulation [cf. [17]]. Taken together, these findings suggest that the emotional modulation of the EPN is critically dependent on the availability of limited processing resources. Previous studies revealed attenuated (or even abolished) emotion processing when attentional resources were directed to locations of explicitly task-relevant stimuli [23,24]. The present data extend these findings to the domain of feature-based attention and provide further evidence for the notion that emotional and task-relevant representations may compete for visual processing resources [20,33]. A possible limitation of the present study is that task load was varied between rather than within experimental blocks. It remains to be determined whether task load similarly interferes with selective emotion processing in less predictable paradigms diminishing task demand expectations. A further point of interest is to state more precisely the locus of interference by using attention tasks targeting either perceptual or cognitive load specifically [32].

The previous analyses suggest that the emotional modulation is not effortless and independent of processing resources. However, the automatic detection and priority processing in terms of attention capture of emotional stimuli may not be distributed uniformly across emotional stimuli but primarily related to evolutionary significant themes [34]. Consistent with this notion, a large array of studies including ERP and fMRI measures demonstrate that stimuli related to avoid predators and other dangers, and reproduction are particularly efficient to capture attentional resources [5-7,10,11]. Therefore, a more conclusive test for the notion of automaticity of emotional processing may be derived by focusing on highly arousing emotional stimuli of evolutionary significance.

**Figure 3:**
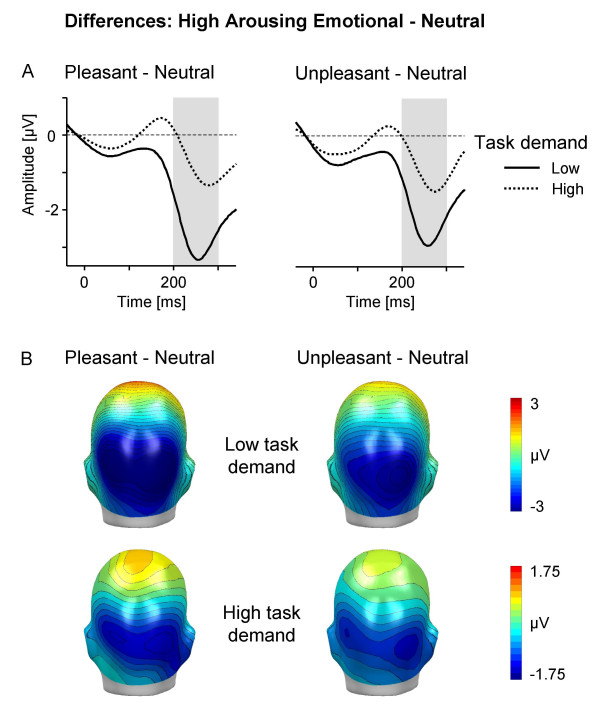
**Interference of high arousing picture processing by task demand**. (A) Difference waveforms (highly arousing emotional – neutral) for low (averaged across the 0 % and 10 % conditions) and high (averaged across the 50 % and 100 % conditions) task demand for a right occipital sensor. The grey-shaded area refers to the analyzed EPN time interval from 200–300 ms. (B) Topographical difference maps for highly arousing pleasant-neutral and highly arousing unpleasant-neutral in low and high task demand conditions (back view; mean from 200–300 ms). Due to the pronounced difference of the emotional modulation for low and high task demand, different scales are used to display the effect.

Accordingly, additional analyses of the emotion-attention interrelationship focused on emotional stimulus materials including only pleasant and unpleasant materials high in emotional arousal in comparison to neutral pictures. Based on the results regarding perceived task demand and the EPN modulation, and to increase signal-to-noise ratio of the ERP, in the present analysis the 0 % and 10 % condition as well as the 50 % and 100 % task conditions were merged representing low and high task demand, respectively. Repeated measure ANOVAs included Valence (highly arousing pleasant, neutral, highly arousing unpleasant), Task (low and high task demand), and Laterality (left, right). The main effect of Valence F(2,30) = 29.5, corrected p < .0001, Greenhouse-Geisser epsilon = 0.80, was qualified by a significant interaction of Valence by Task, F(2,30) = 22.3, corrected p < .0001, Greenhouse-Geisser epsilon = 0.82. To follow up this interaction, low and high task demand conditions were explored in separate ANOVAs. As expected, analysis of the low task demand condition revealed a highly significant main effect of Valence, F(2,30) = 47.0, corrected p < .0001, Greenhouse-Geisser epsilon = 0.84, indicating enlarged occipital negativities for pleasant and unpleasant compared to neutral images, ts(15) < -7, p < .0001, respectively. Interestingly, analysis of the high task demand condition also revealed a significant main effect of Valence, F(2,30) = 5.3, corrected p < .05, Greenhouse-Geisser epsilon = 0.92, although this effect appeared much less pronounced (cf. Figure 3). Specifically, post-hoc tests indicate enlarged EPN for pleasant and unpleasant compared to neutral images, ts(15) < -2.7, p < .05. A further analysis served to statistically secure that high task demands interfered with selective emotion processing. Thus, difference scores of the EPN between emotional (pleasant and unpleasant) and neutral pictures were calculated for low and high task demand conditions, respectively. Repeated measures ANOVA including Task (low and high task demand) and Emotion Difference (pleasant-neutral and unpleasant-neutral picture contents) revealed that the emotional differentiation of the EPN was significantly attenuated in the high compared to low task demand condition, Task F(1,15) = 59.0, p < .0001.

Considering pleasant and unpleasant pictures high in emotional arousal provided a more conclusive test of the notion that the early emotion discrimination occurs automatically. As expected, the data replicated previous studies in that emotional stimuli are particularly efficient to draw attentional resources, i.e., the EPN modulation appeared accentuated for high arousing pleasant and unpleasant compared to neutral stimulus materials [9-11]. Of main interest, corroborating the main analyses, the present findings provide no evidence for the notion of the effortless processing of highly arousing emotional stimuli. Specifically, comparing the magnitude of the EPN modulation of low and high task demand revealed a marked reduction in the emotional modulation for the high demanding task condition (cf. Figure 3). Conceivably, as supported by the significant modulation of the SN and P3b component, the explicit feature-based counting task is drawing processing resources. While attentional resources are scarce, emotional modulation of highly arousing emotional materials is comprised rather than unaffected. A somewhat different perspective of these findings may emphasize that, while markedly attenuated, highly arousing pleasant and unpleasant pictures significantly modulated the EPN during concurrent high task demand. Future studies may determine whether increasing the demands of the primary task can abolish selective emotion processing of high-arousing stimuli. In conclusion, the present findings provide further support for an interference account of the emotion-attention interaction under conditions of competition. From this perspective, selective emotion processing appears as joint function of two opposing factors, the emotional intensity of the emotional cue and the extent of resources demanded by the competing explicit attention task.

**Figure 4:**
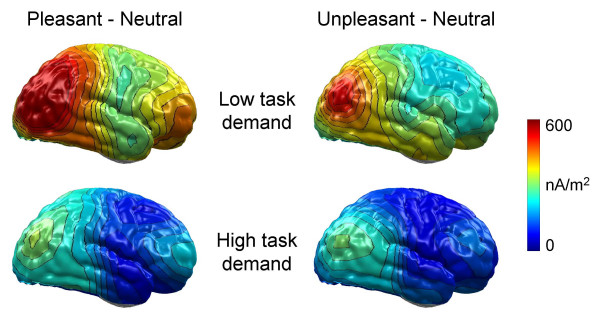
**L2-Minimum-Norm estimates of the emotional modulation**. L2-Minimum-Norm solutions of the EPN effect calculated separately for low (averaged across the 0 % and 10 % conditions) and high (averaged across the 50 % and 100 % conditions) task demand conditions and ERP differences of highly arousing pleasant vs. neutral and highly arousing unpleasant vs. neutral contents. The maps display the right view of a model brain.

A possible concern with regard to the presented analyses is the assessment of emotional modulation in high demanding task conditions. Concurrent performance of a feature-based counting task may impact the topography of scalp-recorded brain potentials. To explore this issue, L2-Minimum-Norm solutions were calculated to provide neural source estimations for the ERP difference potential between emotional (pleasant and unpleasant) and neutral pictures. This distributed source modeling technique requires no a priori assumptions regarding the location and number of current sources [35,36]. Thus, while limited with regard to the accuracy of spatial resolution, this technique appears sufficiently powerful to determine whether emotional cues activate additional brain sources under high compared to low demand task conditions. Focusing on conditions of low task demand, previous results were replicated in that differential processing of highly arousing pleasant and unpleasant contents was modeled primarily by sources over occipito-temporo-parietal regions [9,18]. Interestingly, although much less pronounced, emotional modulation appeared with similar sources over occipito-temporo-parietal regions in high task demand conditions (cf. Figure 4). These results provide further support for the notion that the facilitated processing of emotional cues was attenuated when participants concurrently performed a demanding feature-based counting task.

## Conclusion

From a theoretical perspective, it has been suggested that stimuli are automatically evaluated according to their emotional significance. The notion of automatic affective evaluation implies a collection of features such as being unintentional, effortless, outside of awareness, and linked to approach-avoidance action tendencies [37]. Psychophysiological measures linked to affective response and motor outputs provide evidence for this notion [38-41]. For instance, defensive startle reflex potentiation is observed rapidly after presentation of fear-relevant compared to neutral stimuli in small animal phobia [38]. Moreover, amygdala BOLD signal increases have been observed for threatening facial expressions presented under masking condition presumed to prevent conscious recognition and in blindsight patients [39,40]. The amygdala has been implicated in orchestrating affective learning and may regulate priority processing of emotional cues in the visual cortex [42-44]. These findings provide compelling evidence that selective emotion processing occurs automatically. However, the concept of automaticity needs to be considered not as an all-or-none phenomenon but with regard to separate defining aspects and emotional response measures [19,45]. Suggestive of automaticity, previous research determined that the early emotion discrimination as indexed by the EPN occurs spontaneously, across many stimulus repetitions, and while participants hold task goals in mind [17,18]. In contrast, the present findings suggest that the emotional EPN modulation reflects a capacity-limited process, subject to interference by a competing explicit attention task. Overall, these results appear to support component features rather than all-or-none concepts of automaticity of the early selective emotion processing in the visual cortex [19].

The present findings provide important boundary information regarding the notion of the automatic attention capture of emotional cues during stimulus perception. Taxing processing resources by a competing attention task markedly attenuated the early discrimination of emotional from neutral contents. These results provide further empirical support for an interference account of the emotion-attention interaction under conditions of competition for processing resources. Previous studies revealed the interference of selective emotion processing when attentional resources were directed to locations of explicitly task-relevant stimuli. The present data suggest that interference of emotion processing by competing processing demands is a more general phenomenon extending to the domain of feature-based attention. Accordingly, emotional and task-relevant stimulus representations may be characterized by distinct neural representations, competing for a limited pool of processing resources, potentially attenuating or abolishing selective emotion processing [20,33].

## Methods

### Participants

Participants were sixteen (8 females) right-handed introductory psychology students from the University of Greifswald. Participants were between the ages of 20 and 32 years (M = 22.5). The participants provided written informed consent for the protocol approved by the Review Board of the University of Greifswald.

### Experimental design

Pleasant, neutral, and unpleasant pictures (n = 702) from the IAPS series were presented [25]. The three categories differed significantly from each other in their normative valence ratings (M = 6.8, 5.4, and 2.8 for pleasant, neutral, and unpleasant contents on a 1–9 scale). Mean arousal levels for both emotional categories were significantly higher than for neutral contents (M = 5.2, 3.6, and 5.7 for pleasant, neutral, and unpleasant contents, respectively). To assess the processing of pleasant and unpleasant materials high in emotional arousal, a subset of 100 images for each category was selected based on previous findings [46,47]. High-arousing pleasant materials included primarily scenes of erotica, adventure and sports (Valence M = 6.5; Arousal M = 5.7). High-arousing unpleasant images depicted primarily scenes of mutilations, threat, and violence (Valence M = 2.5; Arousal M = 6.1).

Three out of four experimental conditions included a target counting task. For these conditions, task-related stimuli were created by overlaying six thin horizontal or vertical lines on the IAPS pictures (cf. Figure 1). The probability of task-relevant pictures varied across the three task conditions. The 10 % and 50 % task conditions contained 70 and 351 task-related stimuli, respectively, randomly distributed over the stream of 702 pictures. In the 100 % condition each picture depicted task-related stimuli. In each of the three task conditions, 20 % of the task-related stimuli were targets defined as images depicting either horizontal or vertical lines. Target orientation was balanced across participants. Task performance was intermittently assessed during each task condition. Participants could earn approximately 5 Euro for correct performance in the counting tasks. Furthermore, a passive viewing condition (0 % task) served to replicate previous findings. The order of the four experimental conditions was balanced across participants. Task difficulty was assessed using a 0–100 visual analog scale (ranging from very easy to very difficult) for each of the four conditions.

The pictures were presented in all four experimental conditions as continuous stream without perceivable interstimulus gaps, with each picture shown for 333 ms. Each experimental condition lasted approximately 4 minutes separated by breaks of about 5 minutes between experimental conditions.

### Data collection, reduction, and analysis

Brain and ocular scalp potential fields were measured with a 129 lead geodesic sensor net, ensuring an evenly distributed sensor layout over the head surface with an intersensor distance of about 30 mm. Electrode impedance was kept below 30 kΩ. EEG-data were recorded continuously with the vertex sensor as reference electrode. The data were on-line bandpass filtered from .01 – 100 Hz and sampled at 250 Hz using Netstation software and EGI amplifiers (Electrical Geodesics, Inc., Eugene, Oregon). A 30 Hz low pass finite impulse response (FIR) filter was applied off-line to the continuous EEG data. Stimulus synchronized epochs lasting from 100 ms before until 800 ms after picture onset were extracted. Data editing and artifact rejection were based on a method for statistical control of artifacts [cf. [48]]. First, global artifacts (e.g., due to movements) were detected by analyzing the data after conversion to an average reference and these trials were excluded from further calculations. Second, individual channel artifacts were detected based on the original vertex-referenced data set. Spherical splines [49] weighted on the basis of all remaining sensors served to interpolate artifact-contaminated individual channels on a trial-by-trial base. Finally, signal quality was estimated by calculating the variance of the signal across trials. Data reported are baseline-corrected and converted to an average reference [36]. Emotional modulation of the EPN and attention task effects were explored in two streams of analyses. Accordingly, separate average ERP waveforms were calculated for each experimental condition and the three picture categories for each sensor and participant. In addition, for the 50 % and 100 % task conditions, average ERP waveforms were calculated as a function of task relevance.

#### Selective emotion processing

Based on previous studies, analyses focused on the assessment of the Early Posterior Negativity. This emotional ERP modulation appears as a relative negativity compared to neutral materials across different stimulus materials (e.g., IAPS pictures, emotional faces, words and hand gestures) and experimental protocols associated with distinct ERP topographies [cf. [9-11,13,17,47]] To capture the EPN, the mean activity over a time interval from 200–300 ms was calculated in left and right temporo-occipital sensor clusters (EGI sensor numbers; Left: 58, 59, 60, 61, 63, 64, 65, 66, 67, 69, 70, 71, 72, 74, 75; Right: 77, 78, 79, 83, 84, 85, 86, 89, 90, 91, 92, 95, 96, 97, 100). The emotional modulation appears reversed in polarity over anterior sites. Exploring anterior sensor sites mirrored the effects observed for the occipital negativity and are not reported here for brevity. The relationship of task demand and emotion was further explored by waveform analyses assessing each sensor and time point after picture onset separately [cf. [17]]. The results further substantiated the findings reported here.

#### L2-Minimum-Norm analyses

Calculation of the L2-Minimum-Norm was based on a spherical four-shell isotropic volume conductor head model with 3 (radial, azimuthal, and polar direction) × 197 evenly and spherically distributed dipoles as source model. A source shell radius of 6 cm was chosen as tradeoff between depth sensitivity and spatial resolution. To achieve an appropriate adjustment between the stability of the inverse solution and spatial resolution, a "Tihkonov regularization" was applied to the pseudo-inverse matrix. Specifically, the root mean square of difference between the original and the estimated data (inverse/forward calculation) is plotted across a range of regularization parameters and an optimal "Tihkonov regularization" is empirically determined [L-curve fitting; [35,36]].

#### Explicit attention task

The SN amplitude was scored as mean activity over a time interval from 200 – 300 ms in the same left and right temporo-occipital clusters as the EPN amplitude. The P3b amplitude was scored as mean activity over a time interval from 450 – 550 ms in centro-parietal clusters comprising the following sensors of the EGI net: 7, 31, 32, 38, 43, 53, 54, 61, 60 (left hemisphere), and 79, 80, 81, 86, 87, 88, 94, 106, 107 (right hemisphere). As in other research [50-52], the P3b wave to target pictures was obtained in the presence of the upcoming stimuli. Due to the randomized presentation of IAPS picture categories (pleasant, neutral, and unpleasant), demands of picture processing associated with the subsequent stimuli were kept constant across target and non-target conditions. Thus, the target P3b wave appears to be related to the attention task. In addition, since the P3b component to the target images occurred at a time at which the following picture has already been displayed, these trials were excluded from the main analyses exploring emotional modulation. Thus, contamination of the ERP waveforms to the IAPS pictures by P3b waves to the preceding target stimuli was circumvented.

## Authors' contributions

HTS designed the study, was involved in data analysis, and composed the paper, JS was involved in data analysis and revising the manuscript, FB collected data and contributed to revising the manuscript, MJ developed and accomplished data analyses, AIW helped to design the study and contributed to revising the manuscript, AOH supervised the study and took part in writing the manuscript. All authors read and approved the final manuscript.
